# Ursolic Acid Induces Beneficial Changes in Skeletal Muscle mRNA Expression and Increases Exercise Participation and Performance in Dogs with Age-Related Muscle Atrophy

**DOI:** 10.3390/ani14020186

**Published:** 2024-01-05

**Authors:** Scott M. Ebert, Celine S. Nicolas, Paul Schreiber, Jaime G. Lopez, Alan T. Taylor, Andrew R. Judge, Sarah M. Judge, Blake B. Rasmussen, John J. Talley, Christophe A. Rème, Christopher M. Adams

**Affiliations:** 1 Emmyon, Inc., Rochester, MN 55902, USA; scott-ebert@emmyon.com (S.M.E.); andrew-judge@emmyon.com (A.R.J.); sarah-judge@emmyon.com (S.M.J.); john-talley@emmyon.com (J.J.T.); christopher-adams@emmyon.com (C.M.A.); 2 Division of Endocrinology, Diabetes, Metabolism, and Nutrition, Department of Medicine, and Department of Biochemistry and Molecular Biology, Mayo Clinic, Rochester, MN 55905, USA; 3 Market Unit Petfood Petcare, Virbac SA, 06511 Carros, France; 4 Research & Development—Biopharmacy Department, Virbac SA, 06511 Carros, France; 5 US Petcare Innovation, Virbac NA, Westlake, TX 76262, USA; 6 Innovation, Business Development, Virbac NA, Westlake, TX 76262, USA; 7 Department of Physical Therapy and Myology Institute, University of Florida, Gainesville, FL 32610, USA; 8 Department of Biochemistry and Structural Biology and Center for Metabolic Health, University of Texas Health Science Center, San Antonio, TX 77021, USA

**Keywords:** ursolic acid, dog, canine, skeletal muscle, muscle atrophy, sarcopenia, cachexia, exercise, activity, dietary supplement

## Abstract

**Simple Summary:**

Dogs often experience a significant deterioration in skeletal muscle health and activity when they become older, ill, or injured. Here, we sought to identify a new nutritional approach to maintaining healthy skeletal muscle in dogs. To this end, we developed a novel canine dietary supplement containing ursolic acid, a natural dietary compound that has been shown to be beneficial for skeletal muscle health in non-canine species. We found that dietary supplementation with ursolic acid was safe and well tolerated by dogs. In addition, we found that dietary supplementation with ursolic acid generated numerous beneficial molecular changes in skeletal muscle of older dogs, which was associated with significant improvements in exercise participation and performance. These results identify a new nutritional approach to maintaining skeletal muscle health and activity in dogs.

**Abstract:**

Muscle atrophy and weakness are prevalent and debilitating conditions in dogs that cannot be reliably prevented or treated by current approaches. In non-canine species, the natural dietary compound ursolic acid inhibits molecular mechanisms of muscle atrophy, leading to improvements in muscle health. To begin to translate ursolic acid to canine health, we developed a novel ursolic acid dietary supplement for dogs and confirmed its safety and tolerability in dogs. We then conducted a randomized, placebo-controlled, proof-of-concept efficacy study in older beagles with age-related muscle atrophy, also known as sarcopenia. Animals received placebo or ursolic acid dietary supplements once a day for 60 days. To assess the study’s primary outcome, we biopsied the quadriceps muscle and quantified atrophy-associated mRNA expression. Additionally, to determine whether the molecular effects of ursolic acid might have functional correlates consistent with improvements in muscle health, we assessed secondary outcomes of exercise participation and T-maze performance. Importantly, in canine skeletal muscle, ursolic acid inhibited numerous mRNA expression changes that are known to promote muscle atrophy and weakness. Furthermore, ursolic acid significantly improved exercise participation and T-maze performance. These findings identify ursolic acid as a natural dietary compound that inhibits molecular mechanisms of muscle atrophy and improves functional performance in dogs.

## 1. Introduction

Skeletal muscle atrophy is a highly prevalent condition in dogs that impairs strength, endurance, activity, and overall health [1,2,3]. Some of the most common causes of muscle atrophy in dogs include malnutrition, advanced age, cancer, chronic kidney disease, congestive heart failure, critical illness, osteoarthritis, traumatic joint injuries, a sedentary lifestyle, and obesity [1,2,3]. Because the causes of muscle atrophy are so prevalent, especially in older animals, muscle atrophy is often multifactorial in nature. In addition, by limiting activity, muscle atrophy interferes with exercise and promotes further muscle disuse and a feed-forward process towards more pronounced functional deficits. Unfortunately, despite its broad impact, skeletal muscle atrophy lacks a pharmacologic therapy and cannot be reliably prevented or reversed by current nutritional or physical approaches. Thus, the deterioration of muscle health and function during aging, illness, and injury represents an enormous unmet need in canine health.

The molecular mechanisms of skeletal muscle atrophy are complex and involve multiple signaling pathways that collectively generate numerous changes in skeletal muscle mRNA levels [4,5,6,7,8]. Many of the mRNAs that increase during muscle atrophy encode proteins that promote muscle atrophy and weakness. Conversely, many of the mRNAs that decrease during muscle atrophy encode proteins that are essential for the maintenance of healthy muscle mass and function. The entire collection of mRNAs that increase or decrease in skeletal muscle as it undergoes atrophy is known as an mRNA expression signature of skeletal muscle atrophy. An mRNA expression signature of muscle atrophy is a molecular signature of fundamental mechanistic importance because it captures many of the molecular changes that are causally related to the pathogenesis of muscle atrophy and weakness [9,10]. 

In previous work, we searched for candidate small molecule inhibitors of muscle atrophy by searching for molecules whose mRNA expression signatures negatively correlated to the mRNA expression signatures of two disparate causes of muscle atrophy (prolonged fasting and spinal cord injury) in two species of skeletal muscle (humans and mice) [9,10]. Our screen revealed ursolic acid [9], a naturally occurring pentacyclic triterpene acid that is found in several edible fruits and herbs [11]. The effects of ursolic acid on skeletal muscle were not known, but the finding that ursolic acid’s molecular signature was opposite to the molecular signature of muscle atrophy suggested that ursolic acid might inhibit muscle atrophy. To test this hypothesis, we initially used mouse models and discovered that dietary supplementation with ursolic acid has a previously unrecognized capacity to increase skeletal muscle mass, muscle quality (specific force), muscle strength, and endurance exercise capacity, leading to protection from muscle atrophy during advanced age, fasting, and muscle disuse [9,12,13]. 

Subsequent studies demonstrated that ursolic acid also reduces skeletal muscle atrophy in mouse models of chronic kidney disease [14], cancer [15], and spinal cord injury [16]; improves muscle function, health span, and lifespan in C. elegans and Drosophila [17,18]; and significantly increases strength in humans [19]. Mechanistically, ursolic acid acts directly on differentiated skeletal muscle cells from both humans and mice, where it alters muscle mRNA expression in a way that helps to maintain cellular processes that are critically important for the normal structure and function of skeletal muscle [8,9,13]. Furthermore, as predicted by the systems biology approach that identified it, ursolic acid has broad effects on molecular signaling pathways that control muscle mass and function, including but not limited to inhibition of pro-atrophy (catabolic) signaling through the FOXO, ATF4-C/EBPβ, myostatin, STAT3, and NF-κB signaling pathways [9,12,13,14,15]. 

Although previous studies demonstrated that ursolic acid inhibits molecular mechanisms of skeletal muscle atrophy in other species, the effects of ursolic acid in dogs remained unknown. In the current study, we began to translate ursolic acid to canine health by testing the hypothesis that ursolic acid might inhibit atrophy-associated mRNA expression in canine skeletal muscle, similar to its effects in non-canine species. In addition, to begin to determine whether the molecular signature of ursolic acid in canine skeletal muscle might have functional correlates consistent with improvements in muscle health, we assessed the effects of ursolic acid on secondary outcomes of exercise participation and T-maze performance.

## 2. Materials and Methods

Placebo and ursolic acid dietary supplements—Placebo and ursolic acid soft chews were manufactured in Tampa, FL, USA for the Virbac Corporation and contained proprietary ingredients that improved the oral bioavailability of ursolic acid in dogs and enhanced the palatability of the soft chews for dogs. Placebo soft chews did not contain ursolic acid but were otherwise identical to the ursolic acid soft chews. Ursolic acid soft chews contained 24 mg ursolic acid, in order to provide approximately 2 mg/kg/day to a ≈ 12 kg beagle dog. The chew size and ursolic acid content were designed for dogs weighing less than 18 kg. Ursolic acid was from a proprietary herbal source. The chews contained 13% protein, 1% crude fiber, and 18% fat.

Safety testing of ursolic acid dietary supplement—The study was designed to assess whether the ursolic acid canine soft chew was safe and well tolerated by dogs. The study was conducted according to Virbac procedures and approved by the Virbac Ethical Committee and by the French Authorities. The study involved two groups of healthy adult kenneled beagle dogs, with 4 females and 4 males in each group. Dogs were fed once daily a ration of Veterinary HPM^®^ Adult Dog Neutered (Virbac, Carros, France) dry kibble, during the whole study period. Canned food (Hill’s Prescription Diet Urgent care a/d) was used to facilitate the intake of the chews. Blood and urine were collected from each animal at baseline, prior to the initiation of the study. Animals in the first group served as negative controls and did not receive any ursolic acid canine soft chew. Animals in the second group received five ursolic acid canine soft chews (for a total of 120 mg ursolic acid) once a day for 28 days, via spontaneous oral intake. The five ursolic acid soft chews provided five times the recommended dose of one soft chew per day. Throughout the 28-day study, behavior, general health, food consumption, and stool appearance were assessed every day, and complete physical examinations, including rectal temperature and body weight measurements, were performed once a week. At the conclusion of the 28-day study, blood and urine were collected again from each animal. Blood and urine samples were used to assess the parameters shown in Table 1.

Overall design of proof-of-concept efficacy study—The study was designed to test the hypothesis that ursolic acid might inhibit molecular mechanisms of muscle atrophy in canine skeletal muscle. The primary study outcome was the effect of ursolic acid on atrophy-associated mRNA expression in canine skeletal muscle. Secondary study outcomes were the effects of ursolic acid on exercise participation and T-maze performance. The study facility (CanCog, Toronto, ON, Canada) was accredited by the local Council on Animal Care as meeting the standards of a Good Animal Practice Facility and complied with all local regulations governing the care and use of laboratory animals, including the Ontario Animals for Research Act (RSO 1990, Chapter A.22) and the guidelines of the Canadian Council on Animal Care (CCAC). The protocol was approved by the study facility’s Animal Care Committee before the start of the trial, and all procedures were designed to avoid or minimize discomfort, distress, and pain to the kenneled animals. The study involved 20 female and male beagle dogs who were greater than 7 years of age and had baseline mild or moderate muscle loss as assessed by the WSAVA muscle condition scoring scale (https://wsava.org/wp-content/uploads/2020/01/Muscle-Condition-Score-Chart-for-Dogs.pdf, accessed on 26 October 2023). Additional inclusion criteria included a cooperative disposition, the absence of serious illness as determined by historical health files, and the absence of medications or supplements that could interfere with the objectives of the study. All animals were spayed or neutered. Animals were fed standard commercial kibble for the duration of the study (Purina^®^ ProPlan^®^ All Ages Sport Active 27/17 Chicken & Rice Formula). Canned food (Purina^®^ ProPlan^®^ Adult Complete Essentials™ Chicken & Rice Entrée) was used for dosing and T-maze testing procedures. Baseline assessments included 5 consecutive days of exercise performance and T-maze testing followed by muscle biopsy. After the completion of baseline assessments, animals were randomly allocated to one of two groups (10 dogs/group) that were matched for baseline T-maze performance, sex, body weight, and age. Animals in the first group served as negative controls and received one placebo soft chew once a day for 60 days, via spontaneous oral intake. Animals in the second group received one ursolic acid canine soft chew (24 mg ursolic acid/day) once a day for 60 days, via spontaneous oral intake. The study was blinded to all personnel involved in the investigation, apart from the technicians responsible for dietary supplement assignment and administration. Health observations were performed on a daily basis throughout the study. Animals underwent repeat exercise performance and T-maze assessments during the final 5 days of product administration (days 56–60), and muscle biopsies were repeated on day 60. 

Skeletal muscle biopsies—Subjects were sedated with intravenous medetomidine (0.03 mg/kg) and butorphanol (0.2 mg/kg), and, if needed, anesthesia was induced with intravenous propofol (4 mg/kg to effect). The dorso-lateral cranial thigh over the quadriceps muscle was shaved and surgically prepared according to standard operating procedures. A regional line block (bupivicaine 1 mg/kg) was administered as a local anesthetic proximal to the biopsy site. A 20-gauge needle was used to make a small (2–4 mm), full-thickness skin incision. Through the incision, a biopsy of the quadriceps muscle was taken using a Jorvet SuperCore Biopsy Instrument 14-gauge × 9 cm (with 2 cm throw). The biopsy instrument was passed through the skin incision into the quadriceps muscle at an angle almost parallel to the limb (approximately 5 to 15 degrees) in a proximal to distal direction. After completion, digital pressure was applied to the biopsy site to control any hemorrhage. Vetbond tissue adhesive was used to avoid any prolonged bleeding. Biopsies were performed at a single site, or over two sites, to collect at least 10 mg of muscle tissue, which was snap-frozen in liquid nitrogen and stored at −80 °C. Following the retrieval of a sufficient sample, meloxicam was administered subcutaneously at 0.2 mg/kg and sedation was reversed with an intramuscular injection of atipamezole, volume-matched to medetomidine. 

Analysis of skeletal muscle mRNA expression—Paired quadriceps muscle biopsies (obtained at baseline (day 0) and following 60 days of daily administration of placebo or ursolic acid) from 8 animals from the placebo group and 9 animals from the ursolic acid group were used for the analysis of skeletal muscle mRNA expression. In two animals from the placebo group and one animal from the ursolic acid group, muscle biopsies did not yield RNA of sufficient quality for mRNA analyses.

RNA extraction, library preparation, sequencing, and analysis were conducted at Azenta Life Sciences (South Plainfield, NJ, USA). Total RNA was extracted from fresh frozen canine muscle biopsy samples using the Qiagen RNeasy Plus Universal Mini Kit, following the manufacturer’s instructions (Qiagen, Hilden, Germany). RNA samples were quantified using the Qubit 2.0 Fluorometer (Life Technologies, Carlsbad, CA, USA) and RNA integrity was checked using the Agilent TapeStation 4200 (Agilent Technologies, Palo Alto, CA, USA). RNA sequencing libraries were prepared using the NEBNext Ultra RNA Library Prep Kit for Illumina, using the manufacturer’s instructions (NEB, Ipswich, MA, USA). Briefly, mRNAs were initially enriched with Oligo(dT) beads. Enriched mRNAs were fragmented for 15 min at 94 °C. First-strand and second-strand cDNA were subsequently synthesized. cDNA fragments were end-repaired and adenylated at the 3′ ends, and universal adapters were ligated to cDNA fragments, followed by index addition and library enrichment by PCR with limited cycles. The sequencing library was validated on the Agilent TapeStation (Agilent Technologies, Palo Alto, CA, USA), and quantified using the Qubit 2.0 Fluorometer (Invitrogen, Carlsbad, CA, USA) as well as by quantitative PCR (KAPA Biosystems, Wilmington, MA, USA). The sequencing libraries were clustered on 2 lanes of a flowcell. After clustering, the flowcell was loaded on the Illumina instrument (4000 or equivalent) according to the manufacturer’s instructions. The samples were sequenced using a 2 × 150 bp paired end (PE) configuration. Image analysis and base calling were conducted by the Control software version v2.2.68. Raw sequence data (.bcl files) generated from the sequencer were converted into fastq files and de-multiplexed using Illumina’s bcl2fastq 2.17 software. One mismatch was allowed for index sequence identification. After investigating the quality of the raw data, sequence reads were trimmed to remove possible adapter sequences and nucleotides with poor quality. The trimmed reads were mapped to the Canis lupus familiaris 3.1 reference genome available on ENSEMBL using the STAR aligner v.2.5.2b. The STAR aligner is a splice aligner that detects splice junctions and incorporates them to help align the entire read sequences. BAM files were generated as a result of this step. Unique gene hit counts were calculated by using feature Counts from the Subread package v.1.5.2. Only unique reads that fell within exon regions were counted. After the extraction of gene hit counts, the gene hit counts table was used for downstream differential expression analysis. Using DESeq2, a comparison of gene expression between the groups of samples was performed, and the Wald test was used to generate normalized counts and log2 fold changes. 

Exercise participation and T-maze assessments—At the beginning and end of the study, animals were encouraged (with praise and canned food) to exercise for 6 min (±1 min) in a designated outdoor arena consisting of both gravel and grass areas for five consecutive days. Animals were leashed or harnessed for the duration of the exercise session and exercise was standardized to the greatest extent possible, varying only by individual willingness and functional ability. Functional tests such as the 6-min walk test have been used in other studies to discriminate between diseased and healthy animals [20,21]. In this study, the test was used to tire the dog before the T-maze test and to score the willingness to exercise. Scoring of the interest in exercise is used in approved questionnaires to discriminate diseased dogs, such as in the Liverpool Osteoarthritis in Dogs (LOAD) questionnaire, for example [22]. The first four days of exercise were used to train animals to the protocol and desensitize them to potential biases of novelty and excitation. On the fifth day of exercise, exercise participation was quantitated on a scale of 0–3, with 0 representing no participation and 3 representing enthusiastic participation. Immediately after the exercise, muscular endurance was assessed with the T-maze test. The T-maze consists of a center runway and two side tunnels parallel to the center runway. All dogs were trained on the T-maze task prior to study initiation. Each T-maze session consisted of 20 trials and a 15 s inter-trial interval. Animals traversed the maze through a center aisle containing 2 weave obstacles and returned to the start box through either the right or left arm of the maze, both of which contained a single jump obstacle. The jump height (i.e., high, or low) was determined for each individual animal based on physical ability and remained consistent throughout the study. Travel through either arm of the maze resulted in a food reward and latency to reach the box served as the performance measure. If a subject did not respond to a trial within 30 s, it was recorded as a non-response. Latencies from overtly distracted trials (e.g., prolonged sniffing within the maze) were omitted within the testing software at the discretion of the technician performing the test. The first 10 trials were used to train animals to the protocol and desensitize them to potential biases of novelty and excitation, and the mean latency in trials 11–20 was quantitated.

Statistics—Statistical analyses were performed with GraphPad Prism using the statistical tests described in the figure legends. *p*-values less than 0.05 were considered significant.

## 3. Results

### 3.1. Dietary Supplementation with Ursolic Acid Is Well Tolerated and Safe in Dogs

To begin to translate ursolic acid to canine health, we developed a novel ursolic acid dietary supplement for dogs. The supplement was a canine soft chew that contained 24 mg ursolic acid. The amount of ursolic acid chosen for the soft chew was based on allometric scaling from mouse and human studies [9,12,13,19,23] and oral bioavailability data from dogs, which predicted that 24 mg/day ursolic acid (approximately 2 mg/kg/day) would provide a sufficient amount of ursolic acid for maximal efficacy in beagle dogs.

To assess whether the ursolic acid canine soft chew was safe and well tolerated by dogs, we performed a safety study in which healthy adult dogs received either zero or five ursolic acid soft chews once a day for 28 days. The five ursolic acid soft chews per day provided 120 mg ursolic acid per day, a five-fold excess relative to the predicted efficacious dose. Each group contained eight dogs, with four females and four males. We found that all animals remained healthy throughout the study. In both groups, some animals developed mild and self-limited skin lesions, lameness, ocular discharge, and dental deposits and gingivitis, but there was no relationship of these findings with the ursolic acid canine soft chew. Food consumption and body temperature were unchanged, and body weight slightly increased in both groups, with no difference between the groups (Table 1). There also were no behavioral changes or gastrointestinal side effects related to ursolic acid, including no change in stool appearance. In addition, there were no clinically significant changes in complete blood counts, plasma glucose, serum electrolytes, serum creatinine, blood urea nitrogen, liver function tests, plasma symmetric dimethylarginine, or urine pH or specific gravity, all of which remained within the normal range (Table 1). A very slight but statistically significant increase in blood glucose and MCV was recorded in the control group but all values remained in the physiological range. The slight increase in these parameters was deemed clinically irrelevant and linked to the natural variability of the tests, which can also be observed in the field. These data indicated that dietary supplementation with ursolic acid is safe and well tolerated by dogs, even when provided at a five-fold excess relative to the predicted amount for adequate dietary supplementation.

### 3.2. Dietary Supplementation with Ursolic Acid Inhibits Atrophy-Associated mRNA Expression in Skeletal Muscle of Older Dogs with Age-Related Skeletal Muscle Atrophy

Ursolic acid inhibits atrophy-associated mRNA expression in non-canine skeletal muscle [9,12,13,14]. To test the hypothesis that ursolic might have similar effects in canine skeletal muscle, we conducted a small, randomized, placebo-controlled, proof-of-concept efficacy study in older beagle dogs with mild or moderate muscle loss due to progressive age-related skeletal muscle atrophy (sarcopenia).

Two cohorts of dogs were randomized to receive either one placebo soft chew or one ursolic acid soft chew (24 mg ursolic acid/day) once a day for 60 days. The cohorts were matched for sex (8 females and 2 males per group), age (mean ± SD = 10.6 ± 2.3 years in the placebo group and 10.5 ± 2.4 years in the ursolic acid group), and body weight (mean ± SD = 10.6 ± 1.3 kg in the placebo group and 10.8 ± 1.4 kg in the ursolic acid group). Furthermore, each cohort contained a broad range of advanced ages (8.2 to 13.8 years old in the placebo group, and 8.2 to 13.7 years old in the ursolic acid group), as well as a broad range of body weights (8.8 to 13.3 kg in the placebo group and 9.4 to 13.6 kg in the ursolic acid group). Consistent with the results of the safety and tolerability study, no adverse effects were attributed to the ursolic acid soft chew by the study veterinarians who were closely monitoring the animals. One very old female in the ursolic acid group was removed from the study at day 45 due to anorexia and ascites; however, the study veterinarians independently determined her illness to be related to her advanced age (13.2 years old) and poor baseline health, not ursolic acid.

Prior to the study, all animals were confirmed to have mild or moderate muscle loss due to age-related skeletal muscle atrophy. Because skeletal muscle gene expression varies widely between individuals and is strongly influenced by age, sex, and body weight, among other factors, and because this pilot study had a small sample size with a broad range of ages and body weights and an unequal number of males and females in each cohort, we designed the study to have intrasubject controls, so that we could compare the final mRNA levels to the initial baseline mRNA levels in each animal. Thus, in each animal, we obtained a biopsy from the quadriceps muscle at baseline (day 0) and following 60 days of daily administration of placebo or ursolic acid. We then isolated RNA from the skeletal muscle biopsies, quantitated the levels of atrophy-associated mRNAs, and analyzed intrasubject changes in skeletal muscle mRNA levels in each animal over the course of the 60-day study.

**Figure 1:**
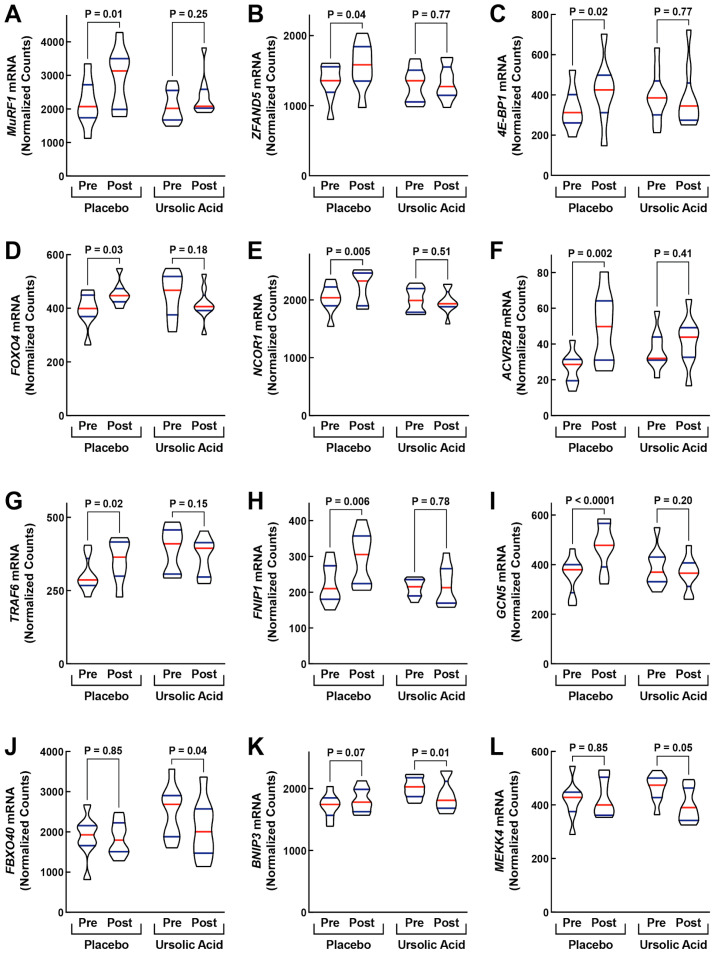
Ursolic acid inhibits atrophy-associated mRNA expression in canine skeletal muscle. Older beagle dogs with mild to moderate age-related skeletal muscle atrophy were randomized to receive either one placebo soft chew or one ursolic acid soft chew (24 mg ursolic acid/day) once a day for 60 days. Quadriceps muscle biopsies were obtained from each animal immediately prior to dietary supplementation (“Pre”) and immediately after 60 days of dietary supplementation (“Post”). RNA was then isolated from the muscle biopsies and used for quantification of mRNAs encoding the muscle atrophy mediators MuRF1/TRIM63 (**A**), ZFAND5 (**B**), 4E-BP1/EIF4EBP1 (**C**), FOXO4 (**D**), NCOR1 (**E**), ACVR2B (**F**), TRAF6 (**G**), FNIP1 (**H**), GCN5/KAT2A (**I**), UBR4 (**J**), BNIP3 (**K**), and MEKK4/MAP3K4 (**L**). Data are truncated violin plots from 8 placebo-supplemented dogs and 9 ursolic acid-supplemented dogs, with red bars denoting median values, blue bars denoting interquartile ranges, and *p*-values determined by two-way ANOVA with multiple comparison testing. MuRF1: Muscle-Specific RING Finger Protein 1; TRIM63: Tripartite Motif Containing 63; ZFAND5: Zinc Finger AN1-Type Containing 5; 4E-BP1: 4E-Binding Protein 1; EIF4EBP1: Eukaryotic Translation Initiation Factor 4E Binding Protein 1; FOXO4: Forkhead Box O4; NCOR1: Nuclear Receptor Corepressor 1; ACVR2B: Activin A Receptor Type 2B; TRAF6: TNF Receptor-Associated Factor 6; FNIP1: Folliculin Interacting Protein 1; GCN5: General Control of Amino Acid Synthesis Protein 5; KAT2A: Lysine Acetyltransferase 2A; UBR4: Ubiquitin Protein Ligase E3 Component N-Recognin 4; BNIP3: BCL2 Interacting Protein 3; MEKK4: MAP/ERK Kinase Kinase 4; MAP3K4: Mitogen-Activated Protein Kinase Kinase Kinase 4.

Importantly, we found that ursolic acid generated numerous changes in mRNA expression that are known to protect the normal structure and function of skeletal muscle and promote overall muscle health. In the skeletal muscle of placebo-supplemented animals, many mRNAs that promote muscle atrophy and weakness exhibited small but significant increases over the course of the 60-day study, consistent with the progressive nature of age-related muscle atrophy. These mRNAs included, but were not limited to, *MuRF1/TRIM63* and *ZFAND5* mRNAs (which encode mediators of muscle protein breakdown and muscle atrophy [24,25,26,27]), *4E-BP1/EIF4EBP1* mRNA (which encodes a protein synthesis inhibitor that negatively regulates muscle mass and function [28]), *FOXO4* and *NCOR1* mRNAs (which encode transcription regulators that negatively regulate protein metabolism and mitochondrial function in skeletal muscle and promote muscle atrophy and weakness [29,30]), *ACVR2B* mRNA (which encodes a receptor for myostatin, a secreted signaling molecule that negatively regulates muscle mass and function [31]), *TRAF6* mRNA (which encodes a mediator of inflammatory signaling that negatively regulates muscle mass and function [32]), *FNIP1* mRNA (which encodes a signaling molecule that inhibits mitochondrial biogenesis in skeletal muscle [33]), and *GCN5/KAT2A* mRNA (which encodes a lysine acetyltransferase that activates NF-κB signaling in skeletal muscle and promotes muscle atrophy [34]) (Figure 1A–I).

**Figure 2:**
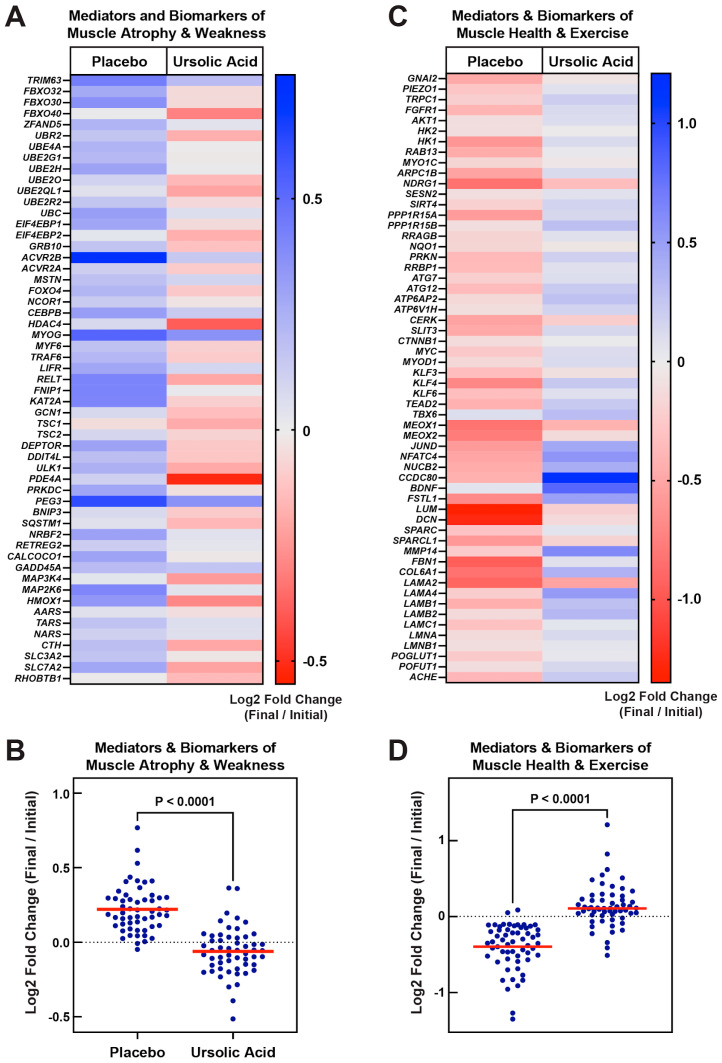
In canine skeletal muscle, ursolic acid decreases mRNAs encoding mediators and biomarkers of atrophy and weakness and increases mRNAs encoding mediators and biomarkers of muscle health and exercise. Older beagle dogs with mild to moderate age-related skeletal muscle atrophy were randomized to receive either one placebo soft chew or one ursolic acid soft chew (24 mg ursolic acid/day) once a day for 60 days. Quadriceps muscle biopsies were obtained from each animal immediately prior to dietary supplementation (“Initial”) and immediately after 60 days of dietary supplementation (“Final”). RNA was then isolated from the muscle biopsies, and log2 fold changes in the indicated mRNA levels were determined in each animal by comparing final mRNA levels to initial baseline mRNA levels in the same animal. Data are from 8 placebo-supplemented dogs and 9 ursolic acid-supplemented dogs. (**A**,**B**) Mean log2 fold changes in mRNAs encoding mediators and biomarkers of muscle atrophy and weakness, shown as a heat map (**A**) and scatter plot (**B**). (**C**,**D**) Mean log2 fold changes in mRNAs encoding mediators and biomarkers of muscle health and exercise, shown as a heat map (**C**) and scatter plot (**D**). In (**B**,**D**), each data point represents the mean log2 fold change in one mRNA, horizontal lines denote means of all assessed mRNA levels, and *p*-values were determined by one-way ANOVA with Dunnett’s multiple comparison tests.

As predicted by its previously described effects in non-canine skeletal muscle, ursolic acid significantly prevented the induction of *MuRF1*, *ZFAND5*, *4E-BP1*, *FOXO4*, *NCOR1*, *ACVR2B*, *TRAF6*, *FNIP1*, and *GCN5* mRNAs in canine skeletal muscle (Figure 1A–I). Ursolic acid also decreased the levels of many other mRNAs that promote muscle atrophy and weakness, including but not limited to *FBXO40* mRNA (which encodes a negative regulator of anabolic signaling and muscle mass [35]), *BNIP3* mRNA (which encodes a mediator of muscle protein breakdown and mitophagy [7,36]), and *MEKK4/MAP3K4* mRNA (which encodes a protein kinase that inhibits mitochondrial function in skeletal muscle and promotes muscle atrophy [37,38] (Figure 1J–L). Altogether, relative to the placebo, ursolic acid significantly reduced or tended to reduce the levels of over 50 mRNAs that encode mediators or biomarkers of muscle atrophy and weakness (Figure 2A,B). Furthermore, the effects of ursolic acid on these pro-atrophy mRNAs suggests inhibition of multiple catabolic signaling pathways that are inhibited by ursolic acid in non-canine skeletal muscle, including but not limited to the FOXO pathway (which includes FOXO4, MuRF1, and BNIP3, among other proteins [7,26]), the ATF4-C/EBPβ pathway (which includes 4E-BP1 and MEKK4, among other proteins [8,13,39]), the myostatin pathway (which includes ACVR2B, among other proteins [28]), the STAT3 pathway (which includes FBXO40, among other proteins [40]), and the NF-kB pathway (which includes GCN5, among other proteins [34]).

In addition to the induction of pro-atrophy mRNAs, skeletal muscle atrophy involves the repression of mRNAs that are important for the normal structure and function of skeletal muscle. In non-canine skeletal muscle, ursolic acid inhibits the overall mRNA expression signature of muscle atrophy, including the repression of pro-atrophy mRNAs and de-repression of mRNA that contribute to the normal structure and function of skeletal muscle. Similarly, in canine skeletal muscle, ursolic acid’s repressive effects on pro-atrophy mRNAs (Figure 1 and Figure 2A,B) were accompanied by the de-repression of numerous mRNAs that encode biomarkers of muscle health and exercise (Figure 2C,D). Collectively, these data indicate that dietary supplementation with ursolic acid inhibits atrophy-associated mRNA expression in canine skeletal muscle and generates a wide spectrum of molecular changes that promote muscle health.

### 3.3. Dietary Supplementation with Ursolic Acid Increases Exercise Participation and T-Maze Performance in Older Dogs

**Figure 3:**
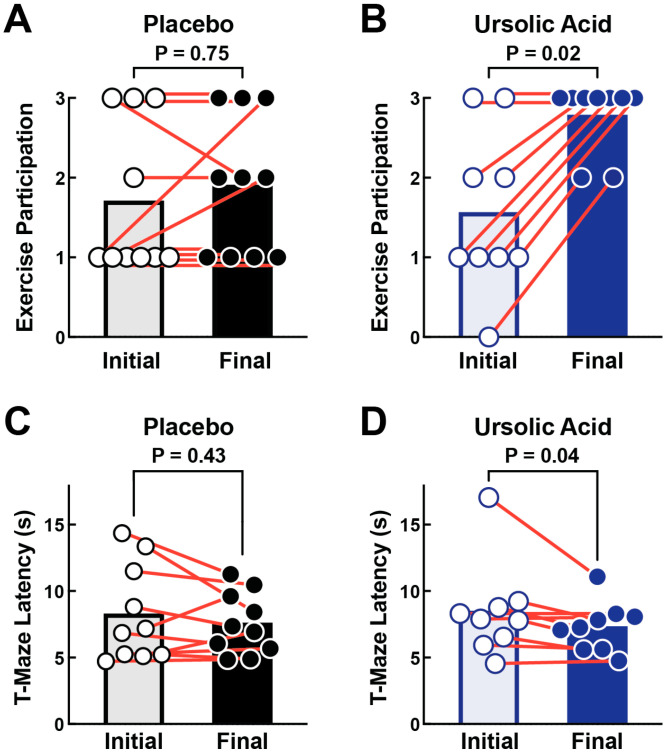
Ursolic acid improves exercise participation and T-maze performance in older dogs. Older beagle dogs with mild to moderate age-related skeletal muscle atrophy were randomized to receive either one placebo soft chew or one ursolic acid soft chew (24 mg ursolic acid/day) once a day for 60 days. (**A**,**B**) Exercise participation was assessed in each animal before and after 60 days of dietary supplementation with placebo (**A**) or ursolic acid (**B**). In this assay, exercise participation is graded on a scale from 0 (no participation) to 3 (enthusiastic participation). (**C**,**D**) T-maze performance was assessed in each animal before and after 60 days of dietary supplementation with placebo (**C**) or ursolic acid (**D**). In this assay, a lower latency score indicates higher T-maze performance. (**A**–**D**) Each data point represents the value from one animal, orange lines denote paired values from the same animal, bars indicate mean values, and *p*-values were determined by two-tailed Wilcoxon matched-pairs signed rank tests.

We hypothesized that the molecular signature of ursolic acid in canine skeletal muscle might have functional correlates consistent with improvements in muscle health. To begin to test this hypothesis, we assessed the effects of ursolic acid on secondary outcomes of exercise participation and T-maze performance, which were determined in each animal at baseline (day 0) and following 60 days of daily administration of placebo or ursolic acid. As expected, in the placebo group, exercise participation did not significantly change over the course of the 60-day study (Figure 3A). In the placebo group, one animal demonstrated a decline in exercise participation during the study, and of the seven animals with impaired exercise participation at baseline, only two showed improved exercise participation over the course of the 60-day study (Figure 3A). In contrast, animals in the ursolic acid group exhibited a striking increase in exercise participation (Figure 3B). In the ursolic acid group, no animals demonstrated a decline in exercise participation during the study, and of the seven animals with impaired exercise participation at baseline, all seven showed improved exercise participation over the course of the 60-day study (Figure 3B). Similarly, T-maze performance did not significantly change in the placebo group (Figure 3C), but it significantly improved in the ursolic acid group (Figure 3D). These data indicate that dietary supplementation with ursolic acid significantly improves activity and functional performance in older dogs, consistent with the molecular effects of ursolic acid in canine skeletal muscle.

## 4. Discussion

In the current study, we sought to identify a new nutritional approach to maintaining healthy skeletal muscle in dogs. To this end, we developed a novel canine dietary supplement containing ursolic acid, a natural dietary compound that inhibits atrophy-associated mRNA expression and improves skeletal muscle health in non-canine species [9,12,13,14,15,16,17,18,19]. Consistent with its effects in non-canine species, dietary supplementation with ursolic acid was safe and well tolerated by dogs. In addition, ursolic acid was strongly efficacious towards the primary outcome of this study, the inhibition of atrophy-associated mRNA expression in canine skeletal muscle. Indeed, in canine skeletal muscle, ursolic acid generated a wide spectrum of molecular changes that contribute to the normal structure and function of skeletal muscle, including the repression of over 50 mRNAs that encode mediators and biomarkers muscle atrophy and weakness, and the de-repression of over 50 mRNAs that encode mediators and biomarkers of muscle health and exercise. Furthermore, the molecular signature of ursolic acid in canine skeletal muscle had two important functional correlates that were consistent with improvements in muscle health, namely increased exercise participation and improved T-maze performance. Collectively, these results identify a new nutritional approach to maintaining skeletal muscle health and activity in dogs. 

We believe that this study had important strengths. First, we rigorously tested the safety and tolerability of the ursolic acid dietary supplement by administering a five-fold higher amount of ursolic acid than is required for adequate dietary supplementation. Second, our proof-of-concept efficacy study was randomized and placebo-controlled, utilized older dogs with age-related muscle atrophy, and employed sensitive molecular assays and intrasubject controls that were well suited for a small proof-of-concept study with wide subject variability. Limitations of the study lie principally in the proof-of-concept efficacy study, which involved a small number of subjects, with an unequal number of males and females and wide variability in ages and body weights. The small sample size and diverse characteristics of the study animals were well suited for a study design that employed intrasubject controls and furthermore allowed us to determine the effects of ursolic acid in animals with diverse characteristics. However, due to the small sample size and diverse characteristics of the study animals, the study lacked adequate statistical power for comparisons of post-treatment outcomes between groups.

It is also important to note that the secondary outcomes in the proof-of-concept efficacy study (exercise participation and T-maze performance) are reflective of muscle health but not entirely specific for muscle health. Indeed, an animal’s enthusiasm and capacity to participate in exercise and to perform well in a T-maze reflect not just healthy skeletal muscle but rather the overall health of the animal, including neuropsychological [41,42,43] and cardiopulmonary function [44], among other factors. Thus, additional studies in the future will be needed to fully understand the histological and physiological consequences of ursolic acid within canine skeletal muscle, and how those changes generate improvements in whole-body functional measures such as exercise participation and T-maze performance. One possibility, which remains to be tested, is that by improving the overall pattern of mRNA expression in skeletal muscle, ursolic acid improves the cellular and physiological function of muscle, which in turn promotes exercise and activity, followed by the well-established beneficial effects of exercise and activity. The current data support a model in which ursolic acid inhibits multiple catabolic signaling pathways in canine skeletal muscle, including the FOXO pathway, the ATF4-C/EBPβ pathway, the myostatin pathway, the STAT3 pathway, and the NF-kB pathway, leading to numerous beneficial changes in skeletal muscle mRNA expression that collectively improve exercise participation and performance.

Despite the current limitations in our understanding of the underlying mechanisms, the observed improvements in exercise participation and T-maze performance clearly indicate a beneficial effect of ursolic acid towards the overall health of animals and suggest the potential for ursolic acid to promote both muscle health and healthy aging in dogs. Furthermore, the improvement in exercise participation may be a particularly meaningful real-world outcome for dogs and pet owners. In addition, the results of this study provide a strong foundation and motivation for longer and more robustly powered trials of ursolic acid, including more comprehensive investigations in dogs with age-related muscle atrophy, as well as additional investigations in dogs suffering from disease conditions that cause muscle atrophy and profound functional deficits, such as cancer, chronic kidney disease, congestive heart failure, critical illness, osteoarthritis, traumatic joint injuries, a sedentary lifestyle, and obesity [1,2,3].

## 5. Conclusions

In conclusion, the current results indicate that ursolic acid is safe, well tolerated, and efficacious as a dietary supplement that promotes the normal structure and function of canine skeletal muscle. These findings identify a new nutritional approach to maintaining healthy skeletal muscle and activity in dogs who are experiencing natural skeletal muscle aging. The results also create an important foundation for future studies focused on additional muscle health outcomes and additional disease conditions that place dogs at high risk for muscle atrophy and functional deficits.

## Figures and Tables

**Table 1:** Dietary supplementation with ursolic acid is well tolerated and safe in dogs. Healthy adult dogs were randomized to receive either no ursolic acid soft chew or 5 ursolic acid canine soft chews (for a total of 120 mg ursolic acid/day) once a day for 28 days. Each group contained 8 dogs (4 females and 4 males). Food consumption, body weight, rectal temperature, complete blood counts, blood chemistry, and urine chemistry were assessed in each animal at baseline and at the conclusion of the 28-day study. Data are means ± SD from 8 animals per condition. Shapiro–Wilk tests were used to assess the distribution of the data. *p*-values were determined with Student’s *t*-tests or Wilcoxon’s signed-rank tests. * *p* < 0.05 vs. initial value. MCV: mean corpuscular volume; MCH: mean corpuscular hemoglobin; MCHC: mean corpuscular hemoglobin concentration; BUN: blood urea nitrogen; AST: aspartate aminotransferase; ALT: alanine aminotransferase; SDMA: symmetric dimethylarginine.

	Control (No Chew)		Ursolic Acid (5 Chews/Day)		Reference Range
	Initial	Final	Initial	Final	
Food consumption (g)	244 ± 55	243 ± 68	275 ± 49	248 ± 77	N/A
Body weight (kg)	8.8 ± 2.8	9.12 ± 2.9 *	9.82 ± 2.8	10.14 ± 3.0 *	N/A
Body temperature (°C)	38.3 ± 0.3	38.4 ± 0.3	38.4 ± 0.3	38.5 ± 0.4	N/A
Complete Blood Counts					
Hemoglobin (g/dL)	16.7 ± 1.6	16.4 ± 1.4	16.1 ± 0.9	16.2 ± 1.2	13.1–20.5
Hematocrit (%)	47.2 ± 4.5	46.2 ± 4.1	45.4 ± 3.3	45.7 ± 3.5	37.3–61.7
Red blood cells (M/µL)	7.45 ± 0.66	7.21 ± 0.64	7.07 ± 0.28	7.09 ± 0.49	5.65–8.87
MCV (fl)	63.3 ± 3.6	64.1 ± 3.5 *	64.1 ± 2.6	64.4 ± 1.9	61.6–73.5
MCH (pg)	22.4 ± 1.1	22.8 ± 1.3	22.7 ± 0.7	22.9 ± 1.0	21.2–25.9
MCHC (g/dL)	35.5 ± 0.5	35.5 ± 0.6	35.5 ± 0.8	35.5 ± 0.8	32.0–37.9
Reticulocytes (K/µL)	21 ± 9	30 ± 8	25 ± 12	28 ± 17	10–110
Platelets (K/µL)	305 ± 69	326 ± 116	277 ± 81	288 ± 90	148–484
White blood cells (K/µL)	8.78 ± 2.10	9.10 ± 3.65	9.16 ± 1.19	8.73 ± 1.56	5.05–16.76
Neutrophils (K/µL)	5.35 ± 1.18	5.90 ± 2.68	5.73 ± 0.81	5.40 ± 1.07	2.95–11.64
Lymphocytes (K/µL)	2.23 ± 0.68	1.96 ± 0.49	2.39 ± 0.67	2.20 ± 0.43	1.05–5.10
Monocytes (K/µL)	0.68 ± 0.27	0.78 ± 0.45	0.69 ± 0.19	0.71 ± 0.31	0.16–1.12
Eosinophils (K/µL)	0.52 ± 0.59	0.46 ± 0.38	0.34 ± 0.35	0.40 ± 0.20	0.06–1.23
Basophils (K/µL)	0.01 ± 0.01	0.01 ± 0.01	0.02 ± 0.02	0.02 ± 0.03	0.00–0.10
Blood Chemistry					
Glucose (g/L)	0.85 ± 0.09	0.95 ± 0.08 *	0.85 ± 0.12	0.86 ± 0.09	0.74–1.43
Sodium (mmol/L)	155 ± 2	155 ± 2	155 ± 2	155 ± 2	144–160
Potassium (mmol/L)	4 ± 0	4 ± 0	4 ± 0	4 ± 0	3.5–5.8
Chloride (mmol/L)	115 ± 2	114 ± 2	115 ± 3	114 ± 2	109–122
Calcium (mg/L)	99 ± 4	99 ± 3	100 ± 5	100 ± 3	79–120
Magnesium (mg/L)	18 ± 1	18 ± 1	18 ± 1	17 ± 1	14–24
Phosphate (mg/L)	38 ± 12	42 ± 4.5	42 ± 8.7	41 ± 8.3	25–68
BUN (g/L)	0.25 ± 0.04	0.26 ± 0.06	0.25 ± 0.07	0.24 ± 0.04	0.15–0.57
Creatinine (mg/L)	6.1 ± 1.3	5.8 ± 1.1	6.6 ± 1.7	6.0 ± 0.9	5.0–18.0
Total protein (g/L)	58 ± 3	59 ± 3	58 ± 2	58 ± 2	52–82
Albumin (g/L)	32 ± 4	33 ± 3	32 ± 1	32 ± 2	23–40
Globulin (g/L)	25 ± 1	26 ± 1	26 ± 1	26 ± 1	25–45
AST (IU/L)	30 ± 8	32 ± 6	31 ± 11	35 ± 9 *	0–50
ALT (IU/L)	48 ± 27	41 ± 11	46 ± 19	46 ± 26	10–125
Alkaline phosphatase (IU/L)	73 ± 25	77 ± 28	104 ± 61	87 ± 68	23–212
SDMA (μg/L)	7 ± 2	7 ± 2	8 ± 2	7 ± 1	0–14
Urine Chemistry					
pH	6.1 ± 0.6	5.7 ± 0.4	6.0 ± 0.4	5.9 ± 0.4	5.3–7.0
Specific gravity	1.040 ± 0.014	1.037 ± 0.010	1.037 ± 0.008	1.032 ± 0.014	1.016–1.060

## Data Availability

The data presented are available on request from the corresponding author.
